# In vivo assembly of the axon initial segment in motor neurons

**DOI:** 10.1007/s00429-013-0578-7

**Published:** 2013-06-02

**Authors:** Barbara Le Bras, Amélie Fréal, Antonny Czarnecki, Pascal Legendre, Erika Bullier, Masayuki Komada, Peter J. Brophy, Marc Davenne, François Couraud

**Affiliations:** 1 INSERM, UMRS 952, 9 Quai St Bernard, 75005 Paris, France; 2 CNRS UMR 7224, 9 Quai St Bernard, 75005 Paris, France; 3 UPMC, Univ Paris 06, 75005 Paris, France; 4 Department of Biological Sciences, Tokyo Institute of Technology, Yokohama, 226-8501 Japan; 5 Centre for Neuroregeneration, University of Edinburgh, Edinburgh, EH16 4SB UK

**Keywords:** Axon initial segment, Development, AnkyrinG

## Abstract

The axon initial segment (AIS) is responsible for both the modulation of action potentials and the maintenance of neuronal polarity. Yet, the molecular mechanisms controlling its assembly are incompletely understood. Our study in single electroporated motor neurons in mouse embryos revealed that AnkyrinG (AnkG), the AIS master organizer, is undetectable in bipolar migrating motor neurons, but is already expressed at the beginning of axonogenesis at E9.5 and initially distributed homogeneously along the entire growing axon. Then, from E11.5, a stage when AnkG is already apposed to the membrane, as observed by electron microscopy, the protein progressively becomes restricted to the proximal axon. Analysis on the global motor neurons population indicated that Neurofascin follows an identical spatio-temporal distribution, whereas sodium channels and β4-spectrin only appear along AnkG^+^ segments at E11.5. Early patch-clamp recordings of individual motor neurons indicated that at E12.5 these nascent AISs are already able to generate spikes. Using knock-out mice, we demonstrated that neither β4-spectrin nor Neurofascin control the distal-to-proximal restriction of AnkG.

**Electronic supplementary material:**

The online version of this article (doi:10.1007/s00429-013-0578-7) contains supplementary material, which is available to authorized users.

## Introduction

The axon initial segment (AIS) constitutes a microdomain in neurons that has emerged as a key structural and functional entity in the last few years. It has been proposed to be responsible for the initiation (Kole and Stuart 2012) or modulation (Zonta et al. 2011) of action potentials. The AIS also contributes to the maintenance of neuronal polarity (Hedstrom et al. 2008; Sobotzik et al. 2009; Rasband 2010): it constitutes a diffusion barrier limiting the mobility of both membrane (Winckler et al. 1999; Nakada et al. 2003) and cytoplasmic molecules (Song et al. 2009).

AnkyrinG (AnkG) (Kordeli et al. 1995), considered as the central AIS organizer, interacts with both the actin cytoskeleton via β4-spectrin (Berghs et al. 2000; Komada and Soriano 2002), and microtubules through the EB3 protein (Leterrier et al. 2011b). AnkG also interacts with extracellular matrix proteins through its direct binding to Neurofascin-186 (Nfasc-186) (Hedstrom et al. 2007). The AIS also contains ion channels. Nav1.1/1.2/1.6 (for a review: Leterrier et al. 2011a), as well as KCNQ2/3 (Kv7.2/7.3) are clustered at the AIS through their direct interaction with AnkG (Garrido et al. 2003; Lemaillet et al. 2003; Pan et al. 2006). Kv1.1/1.2/1.4 (Lorincz and Nusser 2008 *)* and R/T-type calcium channels (Bender and Trussell 2009) are also concentrated at the AIS. The presence of regulatory proteins (Rasband 2010) as well as recent data on activity-dependent plasticity of AIS length or distance from the soma (Grubb and Burrone 2010; Kuba et al. 2010) suggest that AIS structure and role could be more dynamic than originally imagined.

Depletion of AnkG led to the dismantling of AIS proteins in vitro as well as in vivo (Jenkins and Bennett 2001; Hedstrom et al. 2008; Sobotzik et al. 2009). However, in these experiments, the consequences of AnkG loss of expression were assessed after the beginning of axonal development and were therefore linked to a role of AnkG in AIS maintenance rather than AIS assembly.

So far, little is known about the molecular events involved in AIS assembly in vivo and its putative relationship with axonogenesis. Recent data in cultured hippocampal neurons (Sanchez-Ponce et al. 2011) and in *C.elegans* (Maniar et al. 2011) suggested that Ankyrin/UNC-44 is involved in the neuron’s initial polarization through the control of microtubules stability or asymmetry. More recently, a distal sub-membranous axonal cytoskeleton formed by AnkyrinB (AnkB), α2-spectrin and β2-spectrin was shown to restrict AnkG to the proximal part of axons in cortical neurons (Galiano et al. 2012).

To analyze how the AIS initially forms in vivo, we used developing mouse spinal motor neurons (MNs), whose axons are accessible even at their very first stages of development. Our experiments on the global MN population and on single electroporated MNs show that the AIS is formed through mechanisms distinct from those operating in vitro, or in cortical neurons in vivo. In particular, AnkG appears early with Nfasc-186, during axonogenesis, first invading the whole axon; and they only progressively become restricted to its initial segment.

## Materials and methods

### Animals

OF1 mice (Charles River Laboratories, France) were housed under standard laboratory conditions. All animal experiments were performed in compliance with European Community guiding principles on the care and use of animals (86/609/CEE, CE Off. J. no. L358, 18 December 1986), the French decree no. 97/748 of October 19, 1987 (J Off République Française, 20 October 1987, pp. 12245–12248) and recommendations from the CNRS and University Pierre & Marie Curie.

Hb9-GFP transgenic mice in which an eGFP cDNA was expressed under the control of the mouse Hb9 promoter (Wichterle et al. 2002), *β4*-*spectrin* ^−*/*−^ mice lacking the two neuronal isoforms of β4-spectrin, Σ1 and Σ6 (Komada and Soriano 2002) and *Nfasc* ^−*/*−^ mice lacking Nfasc186 and Nfasc155 (Sherman et al. 2005) were used.

### Antibodies

The following primary antibodies were used: mouse monoclonal anti-tau1 (clone PC1C6-MAB3420; IgG2a; Millipore), mouse monoclonal anti-SMI312 directed against phosphorylated neurofilaments (IgG1; Sternberger), mouse monoclonal anti-islet1/2 (IgG1; 39.4D5 and 40.2D6, Hybridoma Bank). Guinea pig and rabbit polyclonal anti-AnkG directed against residues 1633–1650 of human AnkG (Bouzidi et al. 2002) were affinity purified on the peptide and their specificity was assessed by different methods: (i) the labeling with both antibodies was completely suppressed by pre-absorption with the antigenic peptide (Fig. S1 a, b); (ii) the guinea pig, rabbit and mouse monoclonal anti-AnkG (N106/65; IgG2b; Neuromab) antibodies were shown to strictly colocalize at the AISs (Fig. S1 d); (iii) western blots were carried out on proteins extracted from E16.5 spinal cords and protein bands corresponding to 480 and 270 kDa AnkG isoforms were detected whereas no labeling was observed in liver that is known not to express AnkG (Fig. S1 c). Mouse monoclonal (IgG2a) and rabbit polyclonal anti-GFP (Invitrogen), mouse monoclonal anti-PanNav (IgG1; Sigma-Aldrich), rabbit polyclonal NFC2 directed against the intracellular domain of Neurofascin (Tait et al. 2000), mouse monoclonal anti-β4-spectrin raised against residues 2237–2256 of human β4-spectrin (generous gift of M. Solimena), chicken polyclonal anti-β4-spectrin raised against amino acids residues 2171–2345 (Komada and Soriano 2002) and mouse monoclonal anti-α2-spectrin (IgG1; Millipore), mouse monoclonal anti-β2-spectrin (IgG1; BD Biosciences), mouse monoclonal anti-AnkB antibodies (IgG1; N105/17, UC Davies/NIH Neuromab) (Galiano et al. 2012) were used.

Alexa 350-, Alexa 488-, Alexa 594- or Alexa 647-conjugated secondary antibodies (Invitrogen) were used to detect rabbit, guinea pig, chicken polyclonal or mouse monoclonal antibodies.

### Immunohistochemistry

Embryos were collected from pregnant females. Once dissected out of their yolk sac they were immediately immersion fixed in 2 % paraformaldehyde (PFA; in phosphate-buffer saline (PBS), pH 7.4) for 1 h at 4 °C. Embryos were then rinsed, cryoprotected in PBS-20 % sucrose at 4 °C, embedded in OCT medium (VWR) and quickly frozen. Then, 20 μm-thick cryostat serial transverse sections were collected onto slides. Slides were thawed at room temperature (RT) and washed (in Tris-buffered saline (TBS), pH 7.4) and incubated for 1 h in a blocking solution (10 % goat serum in TBS with 0.4 % triton X-100). Slides were then incubated overnight at 4 °C with primary antibodies (diluted in the blocking solution), washed, and incubated for 2 h at RT with the secondary antibodies (diluted in the blocking solution). After washing, slides were mounted in Mowiol medium (Calbiochem). For guinea pig and rabbit anti-AnkG negative controls, 1 μg of peptide with 1 μg of primary antibody was preincubated for 1 h at RT. After whole-cell recordings, the isolated spinal cords were immunostained with the same protocol except for the blocking solution, which contained 0.8 % triton X-100. Images were acquired using a fluorescence microscope equipped with an Apotome module (Zeiss, Axiovert 200 M) and processed with the ImageJ software (Bethesda, MD, USA). Each figure corresponds to a projection image from a stack of optical sections. Panels are oriented with respect to the medio (m), lateral (l) and dorso (d), ventral (v) axes as shown in each figure.

Measures of length and immunofluorescence intensities were performed using the ImageJ analysis software: plot profiles of fluorescence intensity were created for lines drawn along axons in stack-of-interest projected images. After background subtraction, the mean intensity ± s.e.m. from n lines was calculated and shown in the respective images.

### Western blot analysis

Spinal cords and livers were minced and homogenized in ice-cold homogenization buffer (5 mM TrisHCl, pH 7.4, 2 mM EDTA and protease inhibitor mixture consisting of 0.5 mM APMSF with leupeptin, aprotinin, and pepstatin A at 1 μg/ml each). The proteins were then separated on 4.5 % SDS PAGE gel and transferred to nitrocellulose membranes. Membranes were incubated overnight at 4 °C with the rabbit polyclonal anti-AnkG antibody, washed and incubated for 2 h at room temperature with HRP-conjugated anti-rabbit secondary antibodies (Jackson ImmunoResearch). Antibody binding was then visualized by ECL (Amersham).

### Electron microscopy

AnkG localization was analyzed by the pre-embedding immunogold method with silver enhancement (Bernard et al. 1999). Briefly, E11.5 and E13.5 embryos were immersion fixed in PBS with 0.2 % glutaraldehyde and 2 % PFA at RT during, respectively, 30 min and 2 h. E13.5 embryos were further post-fixed in PBS with 2 % PFA at 4 °C during 1 h. After agarose inclusion, 80 μm-thick vibratome coronal sections of the spinal cord at the hindlimb level were immunolabeled with rabbit anti-AnkG antibodies and Fluoro-Nanogold anti-rabbit antibodies (Nanoprobes). The diameter of gold particles was increased using a silver enhancement (HQ silver, Nanoprobes). Sections were then post-fixed, dehydrated and embedded in epon resin. Areas of interest were cut and glued onto resin blocks. 70 nm-thick sections were cut with an ultracut UCT microtome (Leica), stained with lead citrate and analyzed with a transmission electron microscope (EM 912 Omega, Zeiss). Images were captured with a digital camera (SS-CCD, Proscan 1 kx1 k) and analyzed with the iTEM software.

### Mouse embryo electroporation and culture

The electroporation and whole embryo culture protocols were adapted from the ones used by Osumi’s group (Takahashi et al. 2008) to electroporate MNs of E9.5 mouse embryos and to culture them for 24 h. Briefly, E9.5 embryos were dissected out of the uterine wall in Tyrode’s solution. Extra embryonic membranes were removed except the yolk sac and the amnion that were kept intact. Embryos were then transferred into a drop of Tyrode’s solution in a Petri dish filled with sillguard. Plasmid DNA (EndoFree preparation, Qiagen EndoFree MaxiKit) diluted (1 to 1.5 μg/μl) in PBS 1× with Fast Green (0.05 %) was injected with a glass needle and a mouth pipette inside the neural tube. Electrodes (CUY650-P3, Nepagene) were positioned on each side of the embryo. Five pulses (25 V, 50 ms, 1 s interval) were delivered by an electroporator (CUY21SC, Nepagene). Electroporated embryos were transferred to culture bottles on a rotative culture system (BTC Engineering) containing 3 mL of culture medium (100 % rat serum, 10 mM d-Glucose (Sigma-Aldrich), 100U P/S (Sigma-Aldrich)). The culture system was supplied with a gas mixture containing 60 % O_2_-5 % CO_2_. At the end of the culture period, embryos were fixed and frozen as described above, and immunohistochemistry was performed similarly on 50 μm-thick slices.

Quantification of fluorescence intensities along electroporated motor axons was performed using a home-made ImageJ plugin. Axon paths were drawn from three-dimensional image stacks by retrieving the three-dimensional local peak value of GFP fluorescence. Once the path had been retrieved, fluorescence curves along the axon were extracted for each channel of the image stack, i.e., AnkyrinG and GFP. Each path was man checked to handle axon path errors arising from local hot spots in the image stack. The picture of colocalized pixels was obtained by running the ImageJ plugin Colocalization Highlighter on man thresholded GFP stack.

### Electrophysiology

Embryonic spinal cords (SC) from mice were obtained as described previously (Delpy et al. 2008; Scain et al. 2010). Briefly, pregnant mice were anesthetized by intramuscular injection of a mix of ketamine and xylazine. Embryos were removed, and the SC was isolated. Whole SCs were then maintained for 1 h before recording at 32 °C in an artificial CSF (ACSF) containing (in mM): 113 NaCl, 25 NaHCO_3_, 1 NaH_2_PO_4_, 4.5 KCl, 11 glucose, 2 CaCl_2_, and 1 MgCl_2_ continuously bubbled with a 95 % O_2_ - 5 % CO_2_ gaz mixture.

The isolated SC was placed in a recording chamber and continuously perfused at RT with the oxygenated ACSF (1.9 ml/min). Standard whole-cell current-clamp recordings of MNs were made under direct visualization using an infrared-sensitive CCD video camera. MNs were distinguishable by their location in the ventral area. To confirm their MN identity, recorded cells were filled with Neurobiotin (1 mg/ml) and revealed in combination with Islet1/2 immunostaining. Whole-cell patch-clamp electrodes were pulled from thick-wall borosilicate glass using a Brown–Flaming puller (Sutter instrument). The tip of the electrode was fire polished using a microforge (Narishige). Patch-clamp electrodes had resistances of 4–6 MΩ. For current-clamp experiments, the electrodes were filled with a solution containing the following (in mM): 96 KMeth, 34 KCl, 10 HEPES, 4 MgCl_2_, 4 MgATP and 10 EGTA, pH 7.2 [290 mosmol/kg H_2_O]. Events were recorded using a Multiclamp 700B amplifier (Molecular Devices). Data were low-pass filtered (6 kHz) and acquired at 50 kHz on a computer using pClamp9 software.

## Results

### Emergence, extension and restriction of AnkG expression along developing motor axons

Spinal MNs are particularly favorable neurons for undertaking in vivo analysis of AIS assembly because their axons can be observed along their complete length in embryonic spinal cord sections. In the mouse embryo, spinal MNs are generated from E9–E9.5 to E10.5–E11 (Arber et al. 1999). HB9 is a homeodomain transcription factor expressed early in all developing spinal MNs as soon as they exit their last mitosis (Dasen and Jessell 2009). We used Hb9-GFP transgenic mice (Wichterle et al. 2002), which express high levels of GFP throughout the dendrites, soma and axon of all developing MNs. AnkG immunostaining of transverse sections of Hb9-GFP embryos at hindlimb level allowed us to analyze the precise distribution of AnkG in GFP^+^ MNs.

**Fig. 1:**
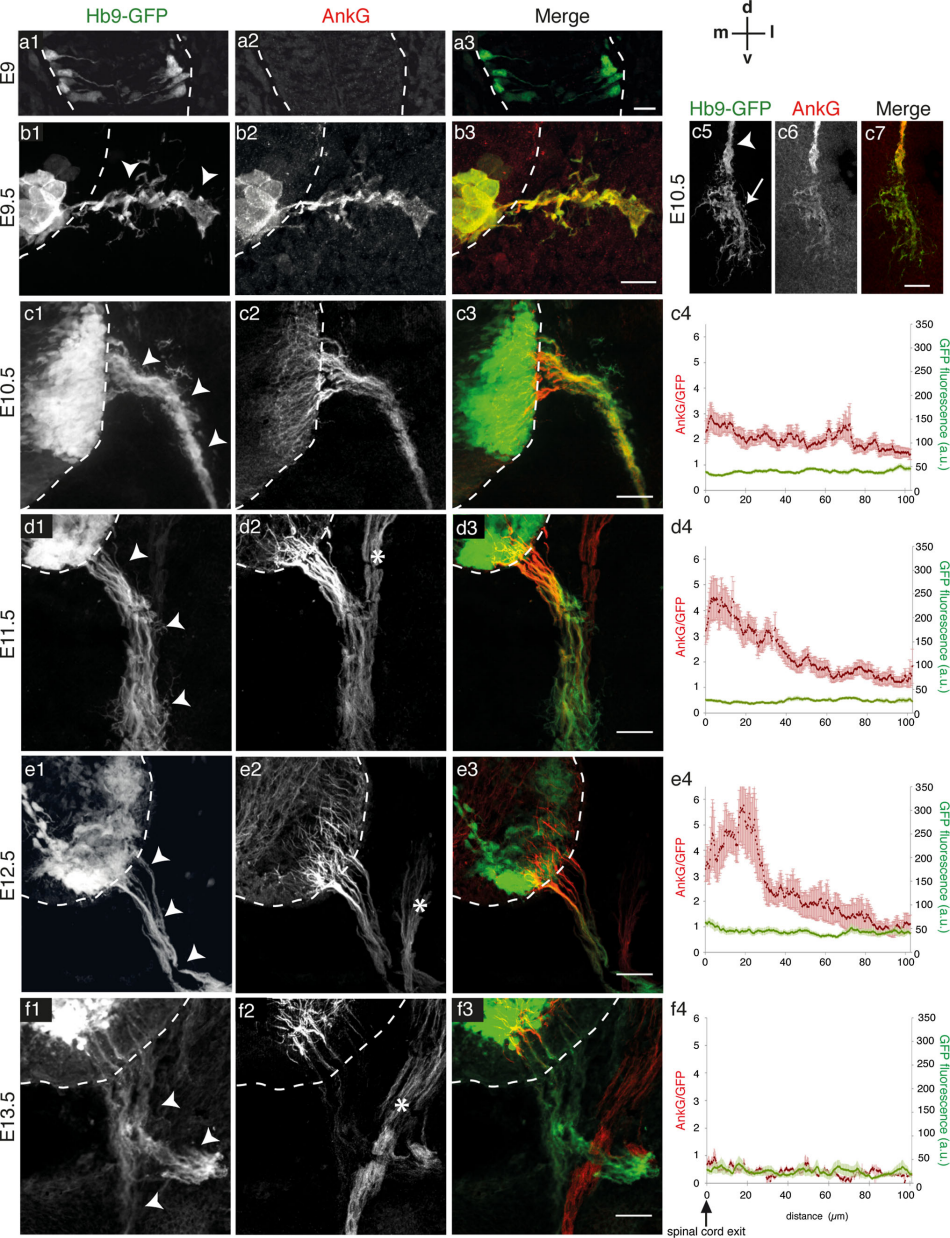
AnkG is first expressed all along the motor axons before being restricted to their proximal part: the AIS. **a**–**f** AnkG immunolabeling (with rabbit anti-AnkG) in the spinal cord (delimited by *dashed lines*) and ventral root (*arrowheads*) in GFP^+^ MNs from Hb9-GFP mouse embryos at E9 (**a**), E9.5 (**b**), E10.5 (**c**), E11.5 (**d**), E12.5 (**e**) and E13.5 (**f**). **a3**–**f3**, **c7** are merged images of AnkG (in *red*) and Hb9-GFP (in *green*). **c4**–**f4** Quantification of AnkG/GFP and GFP fluorescence intensities along the ventral roots (starting from the spinal cord boundary) at the corresponding stage (*n* > 3). *Asteriks* in **d2**, **e2** and **f2** indicate the dorsal root GFP^−^ sensory axons. *Scale bars* represent 15 μm in **a**, 25 μm in **b** and 50 μm in **c**–**f**

At E9, we detected GFP in the soma and processes of a few MNs in the ventral half of the neural tube (Fig. 1a1, the spinal cord is delimited by a dashed line), which had not yet grown an axon exiting the spinal cord. At this stage of development, no AnkG immunostaining could be detected (Fig. 1a2). We could first detect AnkG at around E9.5 in MN cell bodies and axons, depicted as such by their extension out of the spinal cord (arrowheads in Fig. 1b1, Wentworth and Hinds 1978). At this earliest stage of detection, AnkG was expressed along the entire length of axons. Then, from E10.5 AnkG expression decreased in MN somata and was concentrated in their axons (Fig. 1c2). At E10.5, there was a dramatic increase in the number of MNs and their fasciculated axons extended further toward presumptive limb muscles. At this stage, AnkG was again expressed all along axon bundles (arrowheads in Fig. 1c) including in the defasciculating axon terminals (arrow in Fig. 1c5–c7), although AnkG expression was more difficult to track there. AnkG was distributed homogeneously all along axon, as revealed by its constant immunofluorescence (IF) intensity analyzed over the first 100 μm from the spinal cord exit point (as deduced from the globally constant level of AnkG/GFP IF intensity ratio, together with the constant level of GFP IF, Fig. 1c4).

From E11.5 to E13.5 a progressive restriction of AnkG distribution to the proximal part of axon could be observed (Fig. 1d–f). Indeed, a gradual increase of the AnkG/GFP IF intensity ratio occurred in the proximal ventral root (in the first 30–40 μm) relative to its distal part, while GFP IF remained constant all along (Fig. 1d4, e4). In parallel, a progressive decrease of AnkG expression level could be observed in the distal ventral root (Fig. 1d2, e2; the asterisks show AnkG expression in GFP^−^ dorsal root sensory axons). From E12.5 to E13.5 the distal-to-proximal restriction of AnkG expression led to an almost complete absence of AnkG immunostaining in the GFP^+^ ventral root: at E13.5, AnkG had indeed become restricted to axonal segments located only within the spinal cord (Fig. 1f). The mean length of these AnkG^+^ segments was 32.17 ± 1.02 μm (mean ± s.e.m.; *n* = 4; 80 segments), very close to the AIS length measured in adult MNs (30 ± 4.4 μm; Duflocq et al. 2011), suggesting that the size of the AIS may be stabilized very early during MN development. These results thus show that AnkG is first expressed along the entire growing axons of MNs from E9.5 to E10.5, before being restricted to their proximal part, which at E13.5 corresponds in size to the future AIS.

### Fine characterization of AnkG distribution in individual motor axons

**Fig. 2:**
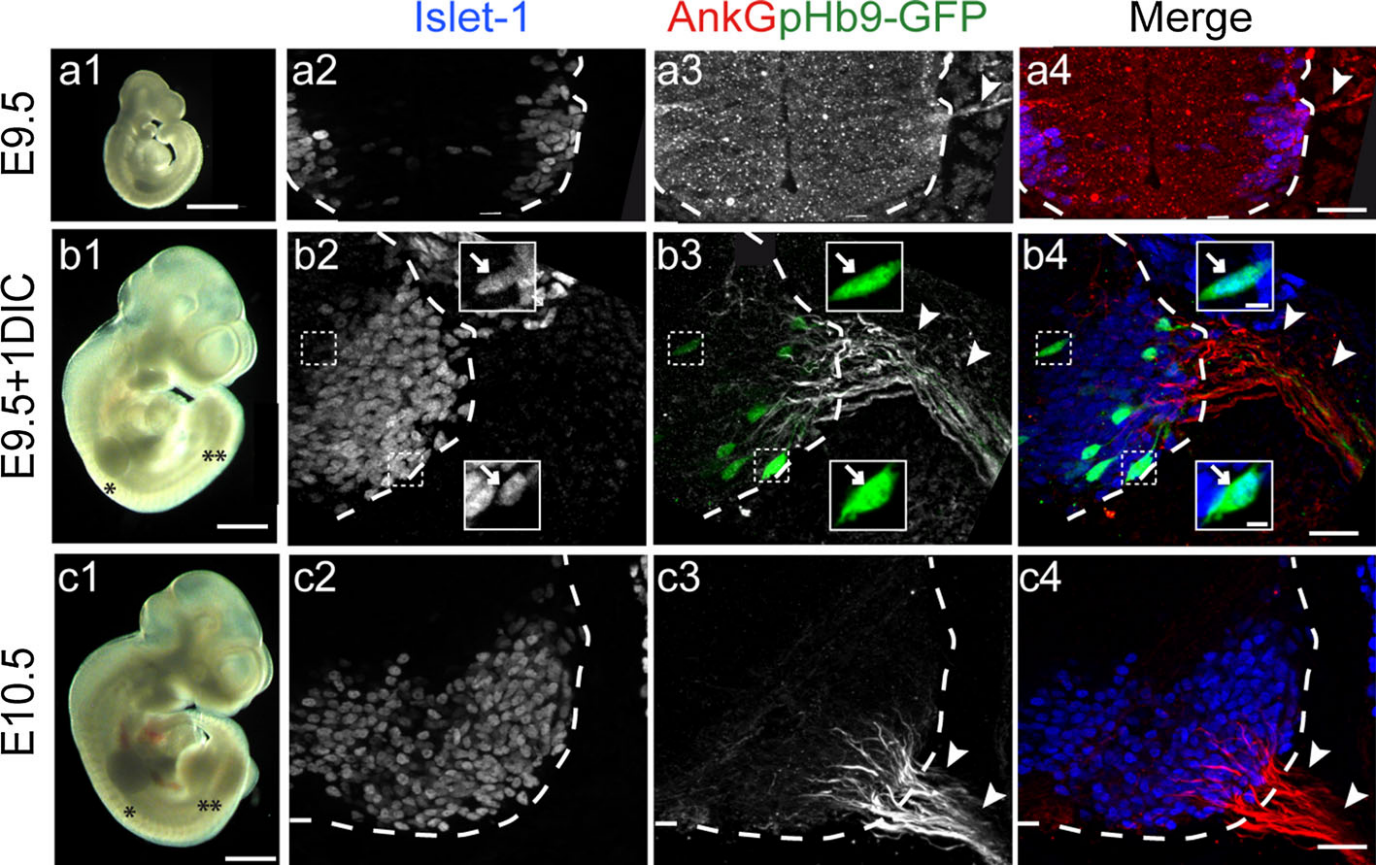
Whole embryos cultured for one day upon electroporation exhibit a normal development. **a1**, **b1**, **c1** E9.5 embryos which were electroporated and then maintained for one day in culture (E9.5 + 1DIC, **b1**) appeared to develop normally when compared to control embryos observed at E10.5 (**c1**) or E9.5 (**a1**): they displayed an increase in their size, the development of forelimb buds (*single asterisk*) and the emergence of hindlimb buds (*double asterisks*). **a2**, **b2**, **c2** Immunolabeling of Islet1 on coronal sections of the spinal cord in (**a1**, **b1**, **c1**) also reveals the increased number of Islet1^+^ MNs in the spinal cord ventral horn after one day in culture (compare **b2**, **c2** and **a2**; *dashed lines* delimit the spinal cord). **a3**, **b3**, **c3** Immunolocalization of AnkG (with rabbit anti-AnkG) confirms the extent of its distribution along developing motor axons (*arrowheads*) in electroporated GFP^+^ MNs as in E10.5 control embryos (compare **b3**, **c3** and **a3** as well as Fig. 1d2). Insets in **b** are enlargements of *dashed boxed* areas, *highlighting* the MN identity of GFP^+^ cells (*arrows*, *n* = 3; 32 neurons). *Scale bars* represent 1 mm in **a1**, **b1**, **c1**, 25 μm in **a2**–**a4**, **b2**–**b4**, **c2**–**c4** and 5 μm in the enlargements in **b**

**Fig. 3:**
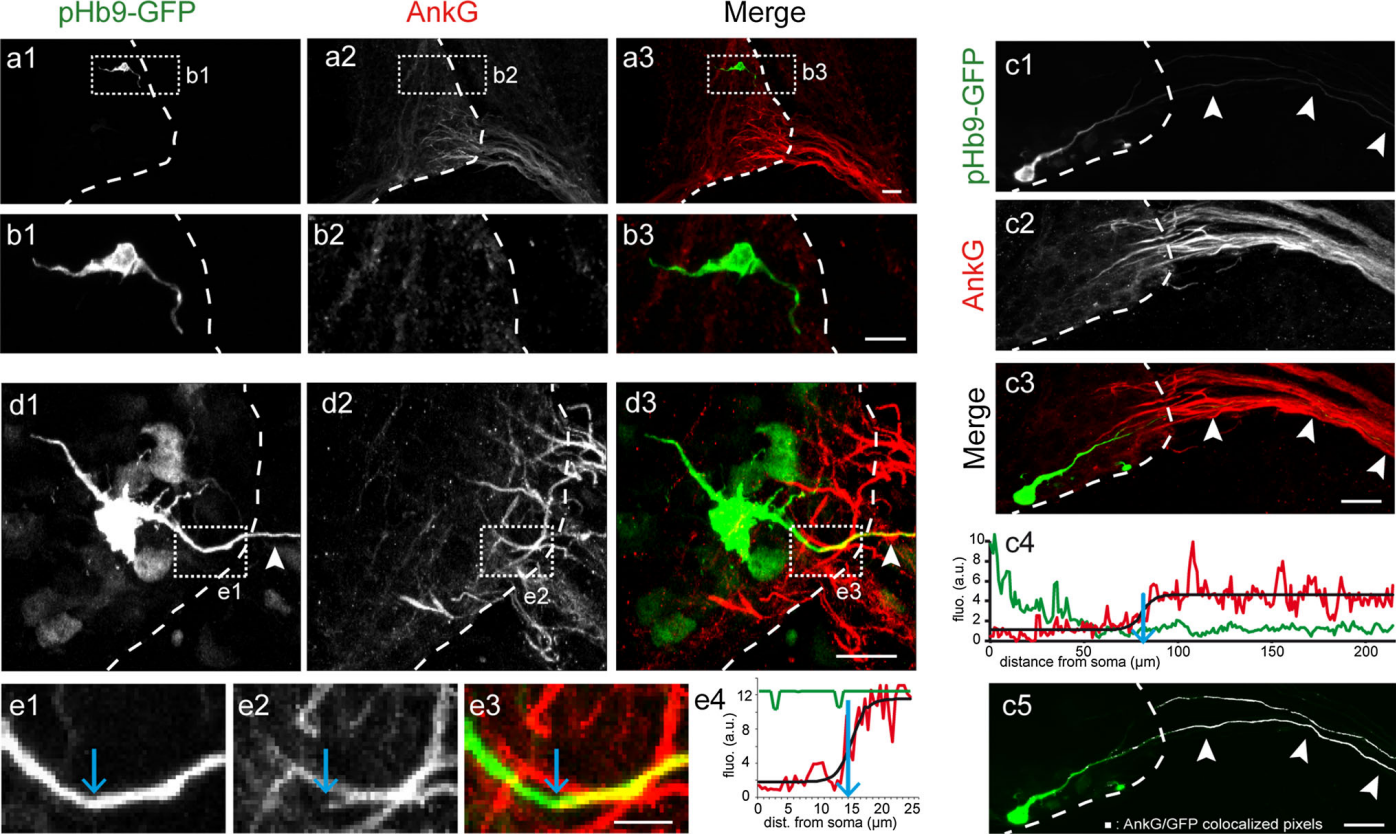
AnkG is never expressed in migrating MNs and its expression is first detected in the axon of morphologically differentiated MNs. **a**, **b** Bipolar MNs electroporated with the Hb9-GFP plasmid did not express AnkG. No AnkG immunolabeling (with rabbit anti-AnkG) (**a2**, **b2**) was detected neither on the trailing nor on the leading process (*n* = 6; 49 neurons) of immature bipolar GFP^+^ MNs (**a1**, **b1**). **b1**–**b3** Enlargements of the *dashed boxes* in (**a1**–**a3**), respectively. **c–e** More differentiated MNs expressed AnkG along their axon. More differentiated GFP^+^ MNs (**c1**, **d1**) with an axon (*arrowheads*) crossing the spinal cord boundary (*dashed lines*) expressed AnkG (**c2**, **d2**). **e1**–**e3** Enlargements of the *dashed box* in (**d1**–**d3**), respectively. **e4**, **c4** Quantification of AnkG (*red*) and GFP (*green*) fluorescence along motor axons shown in **e1** and **c1**, respectively. *Black curves* are theoretical sigmoids fitted to the AnkG fluorescence profile. *Blue arrows* indicate the starting point of AnkG expression, as being the inflexion point of the sigmoid curve. **c5** Distribution of AnkG^+^ pixels (in *white*) colocalizing with GFP (in *green*) in C3, along the GFP^+^ axon (from **c3**). *Scale bars* represent 25 μm in **a**, **b**, **c** and 5 μm in **e**

To analyze more precisely AnkG expression at the level of one single MN, we electroporated E9.5 mouse embryos with an Hb9-GFP coding plasmid (pHb9-GFP, Addgene plasmid 16275, Wilson et al. 2005), and allowed them to grow for one day in culture (1 DIC; Fig. 2a, b). E9.5 electroporated embryos developed well in culture according to macroscopic and microscopic criteria (Fig. 2b): after 1 DIC, embryos exhibited an E10.5 phenotype (compared Fig. 2b, c) as assessed by the appearance of hindlimb buds, the widening of the spinal cord, as well as the increased number of somites, of Islet1^+^ MNs and (as observed in Fig. 1) of AnkG^+^ fibers. Once electroporated, pHb9-GFP was only expressed by MNs, as GFP^+^ cells always coexpressed the pan-MN marker Islet1 (Fig. 2, arrows in b2-4; *n* = 3; 32 neurons). After 1 DIC, electroporated MNs could be found at different stages of differentiation, as revealed by GFP, which labeled either the leading and trailing processes of early post-mitotic MNs migrating toward the peripheral MN pool (Fig. 3a, b), or the axon and dendrites of more mature MNs (Fig. 3c–e). This single-cell labeling approach allowed us to track individual axons within the ventral horn of the spinal cord and sometimes also, although with more difficulty, within the bundle of fasciculated axons in the ventral root. The careful analysis of AnkG immunostaining, in spinal cord transverse sections at hindlimb level, thus allowed us to characterize the distribution of AnkG during the early development of individual MNs.

AnkG immunostaining was never detected in bipolar migrating MNs, neither in their leading nor in their trailing process (*n* = 6; 49 neurons) (Fig. 3a, b). On the contrary, asymmetrical MNs, with a single long process (Fig. 3c) and sometimes multiple much shorter neurites (Fig. 3d), expressed AnkG. In these more mature MNs, AnkG was expressed in one single process only, the longest one, directed toward the ventral horn and exiting the spinal cord, thus identified as the axon (Fig. 3c–e; Wentworth and Hinds 1978). Given the high density of motor axons, the AnkG immunostaining of an individual GFP^+^ axon often overlapped with surrounding AnkG^+^ axons in the 2D projection image (Fig. 3c3, d3). However, the 3D image stack allowed unambiguous tracking of the GFP^+^ axon of interest. The proximal limit of AnkG expression could thus be determined by following AnkG IF intensity along the axon, and by fitting a theoretical sigmoid (Fig. 3c4, e4, black line) to the experimental curve (in red), allowing an unbiased and reproducible determination of the inflexion point that we set as the starting point of AnkG expression (blue arrows). The distance between the soma and the proximal limit of AnkG expression was highly variable, from 9.40 to 81.90 μm (as in Fig. 3c) with a mean value of 35.2 ± 4.56 μm (mean ± s.e.m.; *n* = 5; 19 neurons). Thus, at these early stages of axonal development, a relatively long section of the proximal axon did not express AnkG. Along the entire AnkG^+^ portion of the axon, AnkG displayed a globally constant IF intensity (Fig. 3c4) indicating that there is no gradient or hot spot of AnkG when its expression first appeared, at least within the first 220 μm of axon analyzed, consistent with the results obtained on the global population of MNs at E10.5 (Fig. 1c4).

### AnkG displays a strict sub-membranous localization

**Fig. 4:**
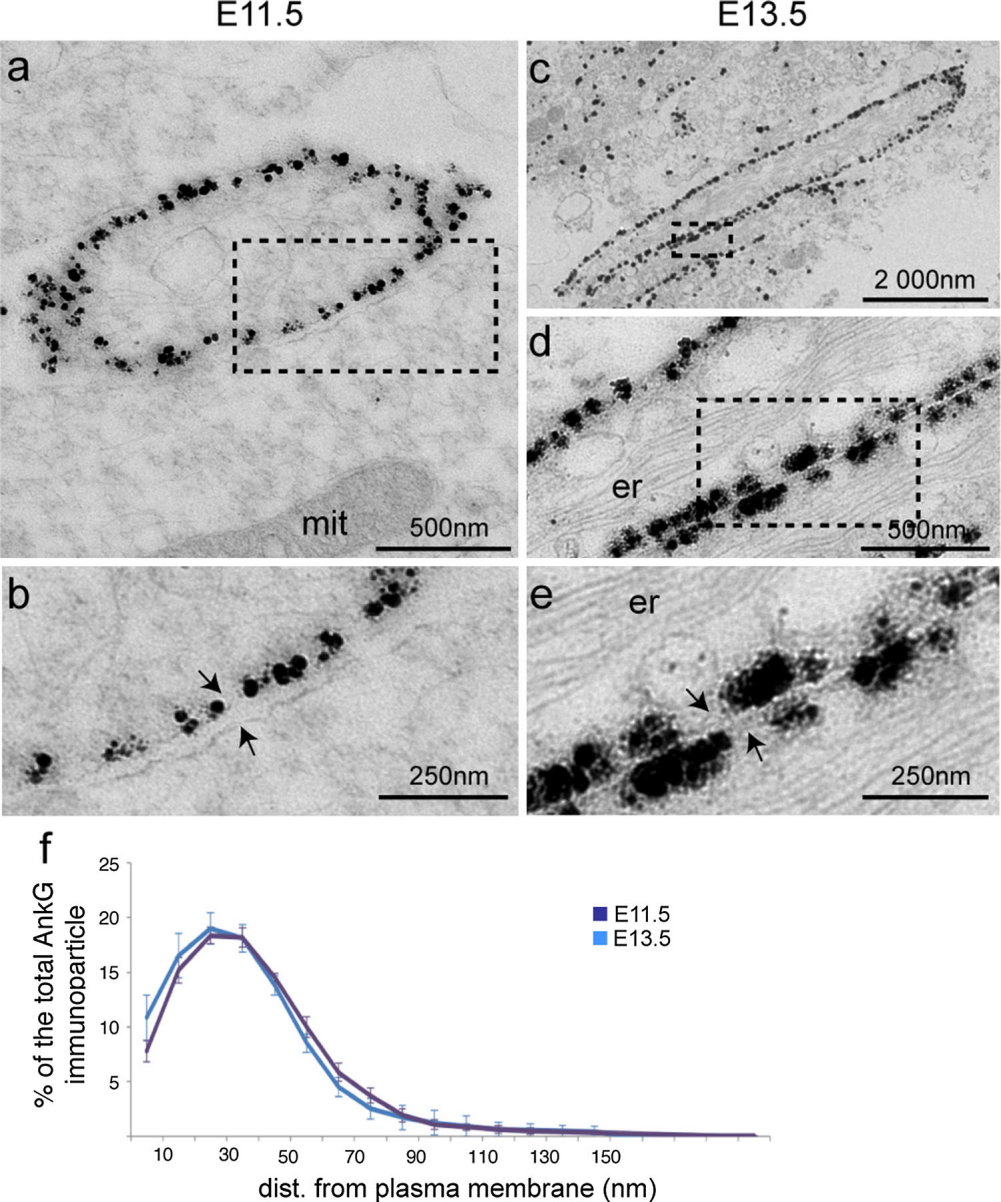
Subcellular distribution of AnkG analyzed by electron microscopy revealed a strict sub-membranous localization. **a**–**e** Subcellular distribution of AnkG immunolabeling (with rabbit anti-AnkG) in ultrathin sections from E11.5 (**a**, **b**) and E13.5 (**c**–**e**) ventral spinal cords (*arrows* in **b** and **e** point to the plasma membrane). **b**, **d** and **e** represent an enlargement of *dashed boxes* in **a**, **c** and **d**, respectively. **f** Distribution of AnkG immunoparticles with respect to the distance from the plasma membrane at E11.5 (*purple curve*) and E13.5 (*blue curve*). No difference could be found between these two stages (two-tailed unpaired *t* test, *p* > 0.05, *n* = 2). *mit* mitochondria, *er* endoplasmic reticulum. *Scale bar* represent 2 μm in **b**; 500 nm in **a**, **c** and 250 nm in **b**, **e**

To further characterize, at a subcellular level, AnkG distribution in individual axons during AIS assembly, we analyzed by electron microscopy AnkG immunolabeling in axonal tracts from the spinal cord ventral horn. At E11.5, a clear labeling was found just beneath the membrane only (Fig. 4a, b, arrows point to two adjacent plasma membranes). The distance from the membrane of AnkG immunolabeling was measured for all gold particles, whose surrounding membranes could be identified (Fig. 4f). More than 90 % of these immunoparticles were located in the first 60 nm underneath the plasma membrane. We also analyzed AnkG subcellular distribution at E13.5 (Fig. 4c–e). There was no difference in AnkG distribution between these two stages (Fig. 4f, comparison every 10 nm: unpaired two-tailed *t* test, *p* > 0.05, *n* = 2; at least 5 sections). Thus, AnkG is already concentrated just beneath the plasma membrane at a stage when it is distributed all along the axon and starts being restricted to the proximal part of the axon (E11.5). The same sub-membranous distribution was found at E13.5, when AnkG was restricted to nascent AIS. If the immunolabeling conditions allowed us to detect plasma membranes and mitochondria, they were not suitable for the observation of the actin and/or microtubule cytoskeletons. Recent data obtained in the cerebellar cortex showed that in the AIS of adult neurons, AnkG also exhibited a sub-membranous localization (Iwakura et al. 2012). Our results suggest that at least some of the mechanisms that ensure the final localization of AnkG right beneath the membrane are already functional at this very early stage of AIS assembly.

### Sodium channels appear after AnkG, at E11.5, and start generating action potentials one day later

**Fig. 5:**
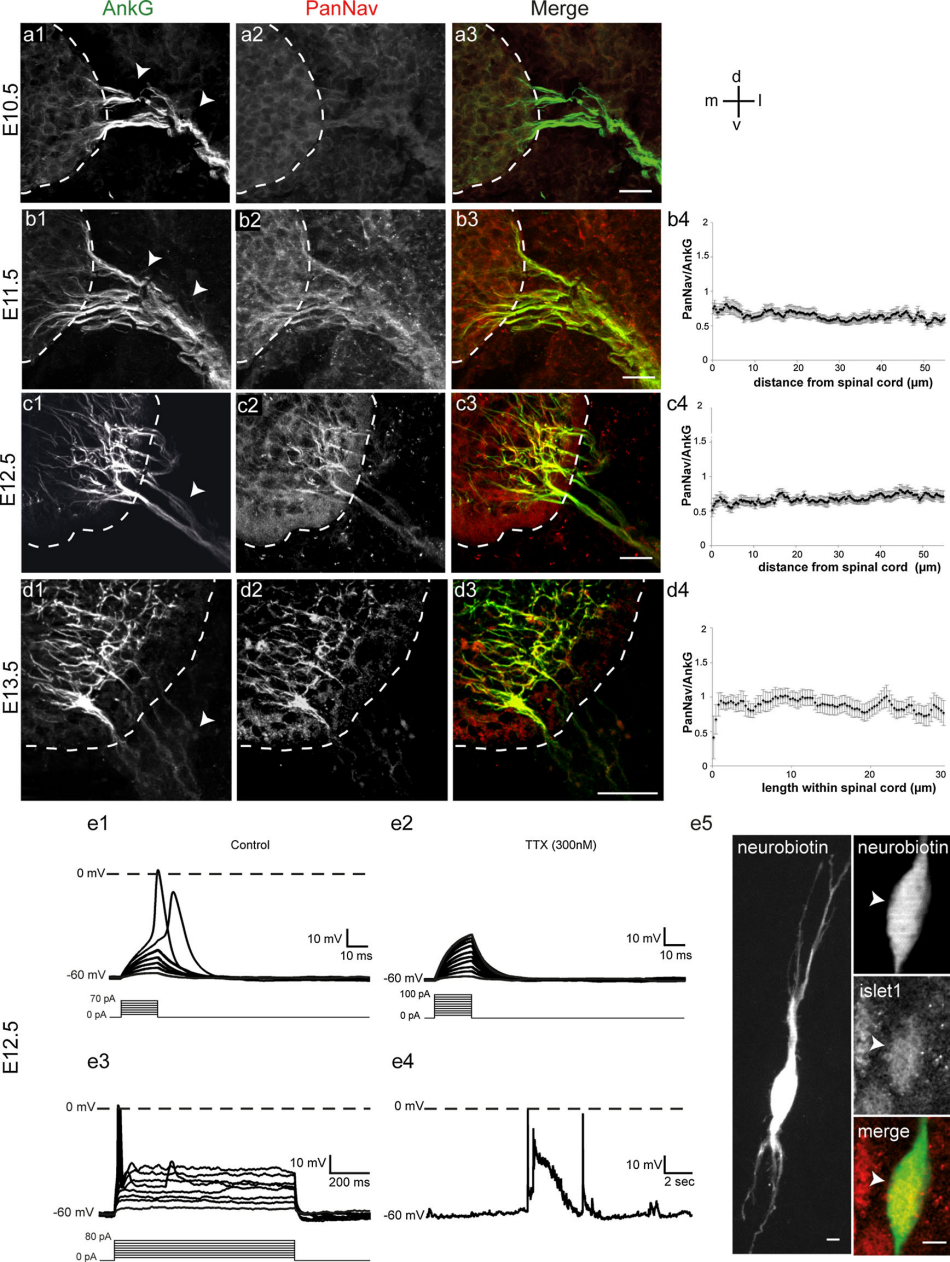
Nav channels start being expressed after AnkG, at E11.5, and MNs are excitable at E12.5. **a**–**d** Nav are expressed from E11.5 in the MNs’ axons and colocalize with AnkG. Immunolabeling of AnkG (with rabbit anti-AnkG) (**a1**–**d1**) and of all Nav α isoforms (PanNav) (**a2**–**d2**) inside the spinal cord (delimited by *dashed lines*) and in ventral roots (*arrowheads*) at E10.5 (**a**), E11.5 (**b**), E12.5 (**c**) and E13.5 (**d**). **a3**–**d3**) Are merged images of AnkG (in *green*) and PanNav (in *red*). **b4**, **c4**, **d4** PanNav/AnkG IF intensities along AnkG^+^ fibers in ventral roots at E11.5 (**b4**) and E12.5 (**c4**) and in the spinal cord at E13.5 (**d**4) (*n* = 3). **e** Firing properties of E12.5 lumbar MNs (**e1**) Short-duration (20 ms) depolarizing currents (10 pA incremental current steps) were injected to generate single action potentials. **e2** Action potentials generation was totally blocked in the presence of TTX (300 nM). **e3** A prolonged (800 ms) current injection generated only 1 action potential. **e4** Spontaneous activity in E12.5 MNs. All current-clamp recordings were performed from a holding potential of −60 mV. **e5** The neuron filled with neurobiotin during the recording was immunostained with Islet1 to confirm its MN identity. Scale bars represent 25 μm in **a**–**d** and 5 μm in **e**

Nav channels are highly enriched at the AIS of mature neurons and are responsible for initiating and/or modulating action potentials. Their direct interaction with AnkG is necessary for their accumulation at the AIS (Garrido et al. 2003; Lemaillet et al. 2003). Thus, as a first step toward elucidating the hierarchy between Navs and AnkG in AIS assembly in vivo, we analyzed their relative expression patterns during MN development. We used a PanNav antibody that specifically recognizes all Nav α-subunits. At E10.5, no Nav expression could be detected neither in the spinal cord nor in the ventral root, while AnkG was already robustly expressed along growing motor axons (Fig. 5a). At E11.5, a weak labeling of Navs could be detected along all AnkG^+^ fibers both within the spinal cord and along the ventral root (Fig. 5b). The labeling of Navs strictly co-localized with that of AnkG along the entire length of AnkG^+^ fibers. At E12.5 (Fig. 5c) and E13.5 (Fig. 5d), the PanNav labeling still colocalized with AnkG labeling, being like AnkG progressively restricted to the proximal part of axons inside the spinal cord. We also measured, from E11.5 to E13.5, the ratio between Nav and AnkG IF intensities to assess whether the two proteins not only co-localized but also displayed proportional expression levels all along AnkG^+^ fibers. From E11.5 to E13.5, the PanNav/AnkG ratio was always constant all along AnkG^+^ axons (Fig. 5b4, c4, d4). This indicates that even though Navs appeared after AnkG they always displayed the same distribution as AnkG along motor axons, with a progressive proximal restriction (as seen in Fig. 1), such that the amount of Navs relative to AnkG proteins was homogeneous along motor axons.

To test whether these Nav channels were already functional (i.e., able to generate spikes) at these early stages of Nav expression and motor axon development, we used whole-cell patch-clamp recordings, in the current-clamp configuration, of MNs at E12.5, i.e., 24 h after the emergence of Nav protein expression. Short-duration (20 ms) depolarizing currents (10 pA incremental current steps) induced the generation of single action potentials that were abolished by the addition of 300 nM tetrodotoxin (Fig. 5e1, e2), indicating that at this early stage Navs were able to generate spikes. A prolonged (800 ms) current injection was unable to elicit repetitive firing (Fig. 5e3) in contrast to what can be observed in more mature MNs (Gao and Ziskind-Conhaim 1998). Finally, E12.5 MNs were also found to display a spontaneous spiking activity (Fig. 5e4). This indicates that at this early stage of development, just one day after Navs are first expressed MNs are already excitable, even before they formed connections with muscles (Pun et al. 2002). After recording cells were filled with neurobiotin and their MN identity was confirmed by Islet-1 immunostaining (Fig. 5e5).

### Nfasc is expressed concomitantly with AnkG but is not required for AnkG restriction

**Fig. 6:**
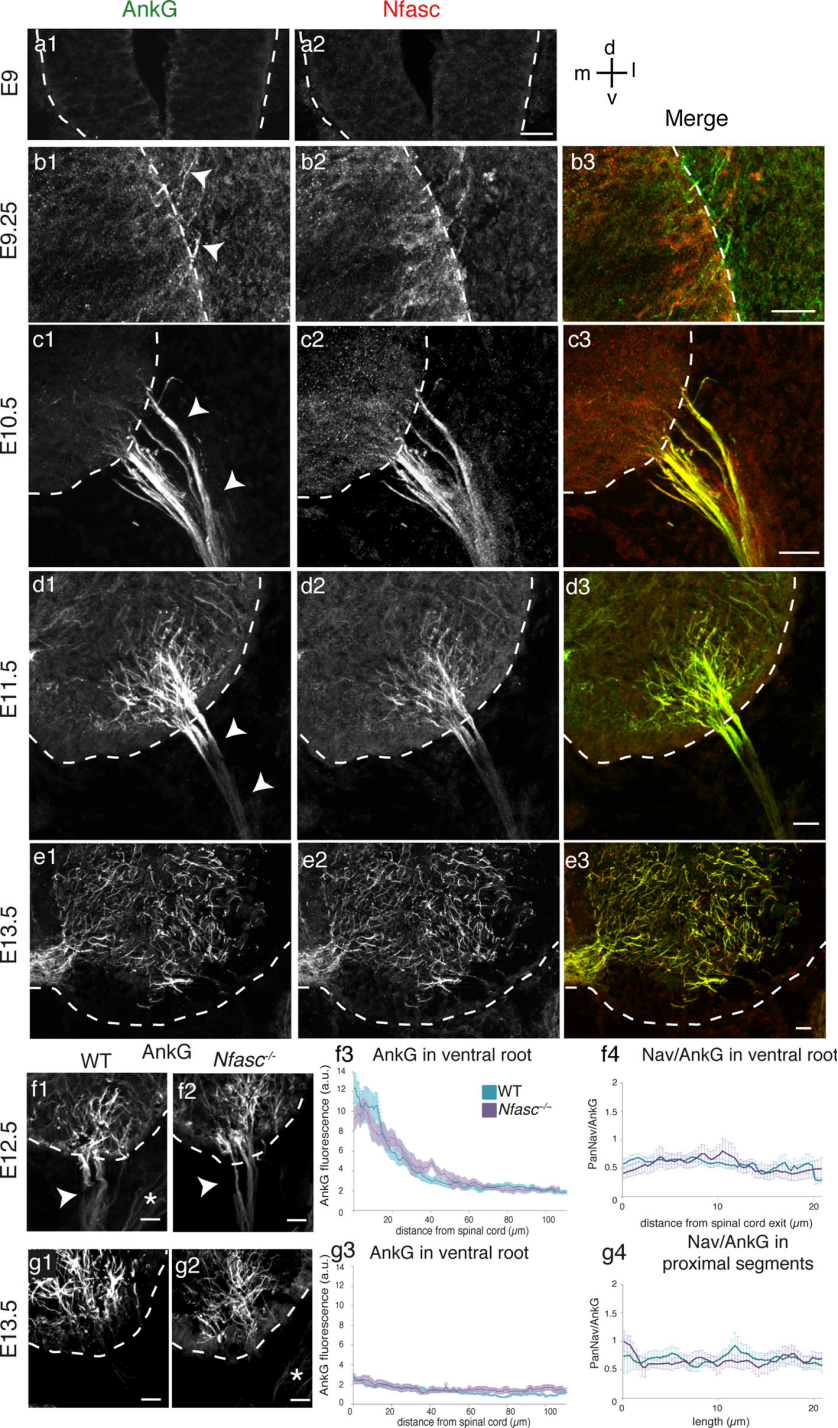
Nfasc follows the same spatio-temporal distribution as AnkG, and its loss of expression does not affect the restriction of AnkG and Navs to the nascent AIS. **a**–**e** Nfasc (**a2**–**e2**) and AnkG (with guinea pig anti-AnkG) (**a1**–**e1**) immunolabelings in the ventral spinal cord (delimited with *dashed lines*) and in the ventral root (*arrowheads*) at E9 (**a**), E9.25 (**b**), E10.5 (**c**), E11.5 (**d**), and E13.5 (**e**). **f–g** AnkG immunolocalization (with guinea pig anti-AnkG) in the spinal cord and in the ventral root of *Nfasc* ^−*/*−^ (**f2**, **g2**) and WT (**f1**, **g1**) embryos at E12.5 (**f1**, **f2**) and E13.5 (**g1**, **g2**). AnkG IF profile along the ventral root (**f3**, **g3**, *n* = 3) and PanNav/AnkG IF intensities (**f**4 in the ventral root and **g4** within the spinal cord, *n* = 3; at least 16 paths measured) in WT or *Nfasc* ^−*/*−^ embryos at E12.5 (**f3**, **f4**) and E13.5 (**g3**, **g4**). *Scale bar* represent 25 μm

Loss-of-function experiments with shRNAs in cultured hippocampal neurons suggested that the neuronal isoform of Nfasc, Nfasc186, is not required for the in vitro assembly or maintenance of the AIS (Dzhashiashvili et al. 2007; Hedstrom et al. 2007). More recent data confirmed in vivo that Nfasc186 was not required for AIS assembly in cerebellar Purkinje cells, however, Nfasc186 was required for AIS maintenance (Zonta et al. 2011). We thus wondered if Nfasc was involved in the first stages of in vivo AIS formation in MNs. We first analyzed the expression pattern of Nfasc by immunostaining with an antibody that recognizes both its glial (Nfasc155) and neuronal (Nfasc186) isoforms. At E9, no immunoreactivity for AnkG nor for Nfasc could be detected in the spinal cord (Fig. 6a). At E9.25, Nfasc started to be expressed in a few MNs and along their axons extending out of the spinal cord (arrowheads, Fig. 6b2). Nfasc expression appeared concomitantly and colocalized with AnkG expression. Nfasc then followed the same spatio-temporal distribution as AnkG: it was first expressed, until E10.5, along the entire length of axons (Fig. 6c), up to axon terminals (data not shown), and then started to display a distal-to-proximal restriction (Fig. 6d), until E13.5, when Nfasc^+^ segments were confined to the spinal cord (Fig. 6e).

Given the early emergence of Nfasc expression, we investigated whether Nfasc plays a role in the early steps of AIS assembly, in particular in the restriction of AnkG and Navs to the nascent proximal AIS. We thus first compared AnkG distribution along the ventral root in *Nfasc* ^−*/*−^ and wild-type (WT) mice at E12.5 (Fig. 6f1–f3) and E13.5 (Fig. 6g1–g3). We found the same distribution profile in both mice, with AnkG being concentrated in the proximal ventral root at E12.5 and absent from the ventral root at E13.5 because of its restriction to proximal axonal segments within the spinal cord at E13.5. These results suggest that AnkG follows a normal restriction despite the absence of Nfasc.

We also compared the PanNav to AnkG IF ratio along the ventral root at E12.5 (Fig. 6f4) and along proximal axonal segments in the spinal cord at E13.5 (Fig. 6g4). We found again the same profile in *Nfasc* ^−*/*−^ and WT mice (student unpaired *t* test *p* > 0.05), suggesting that the amount of Navs relative to AnkG proteins is similar in both WT and mutant mice and constant along these axonal domains, suggesting that the clustering of Navs along AnkG^+^ ventral roots and proximal axons is not affected by the absence of Nfasc.

Hence, despite its early expression, Nfasc does not seem to play a role in the distal-to-proximal restriction of AnkG and Navs, which leads to the assembly of the early functional MN AIS in vivo.

### β4-spectrin is not necessary for early AIS assembly

β4-spectrin is specifically expressed at the AIS where it interacts with both AnkG and actin (Berghs et al. 2000; Komada and Soriano 2002); it may play a key role during AIS assembly. We therefore analyzed the time course and distribution of β4-spectrin expression in the developing MN.

**Fig. 7:**
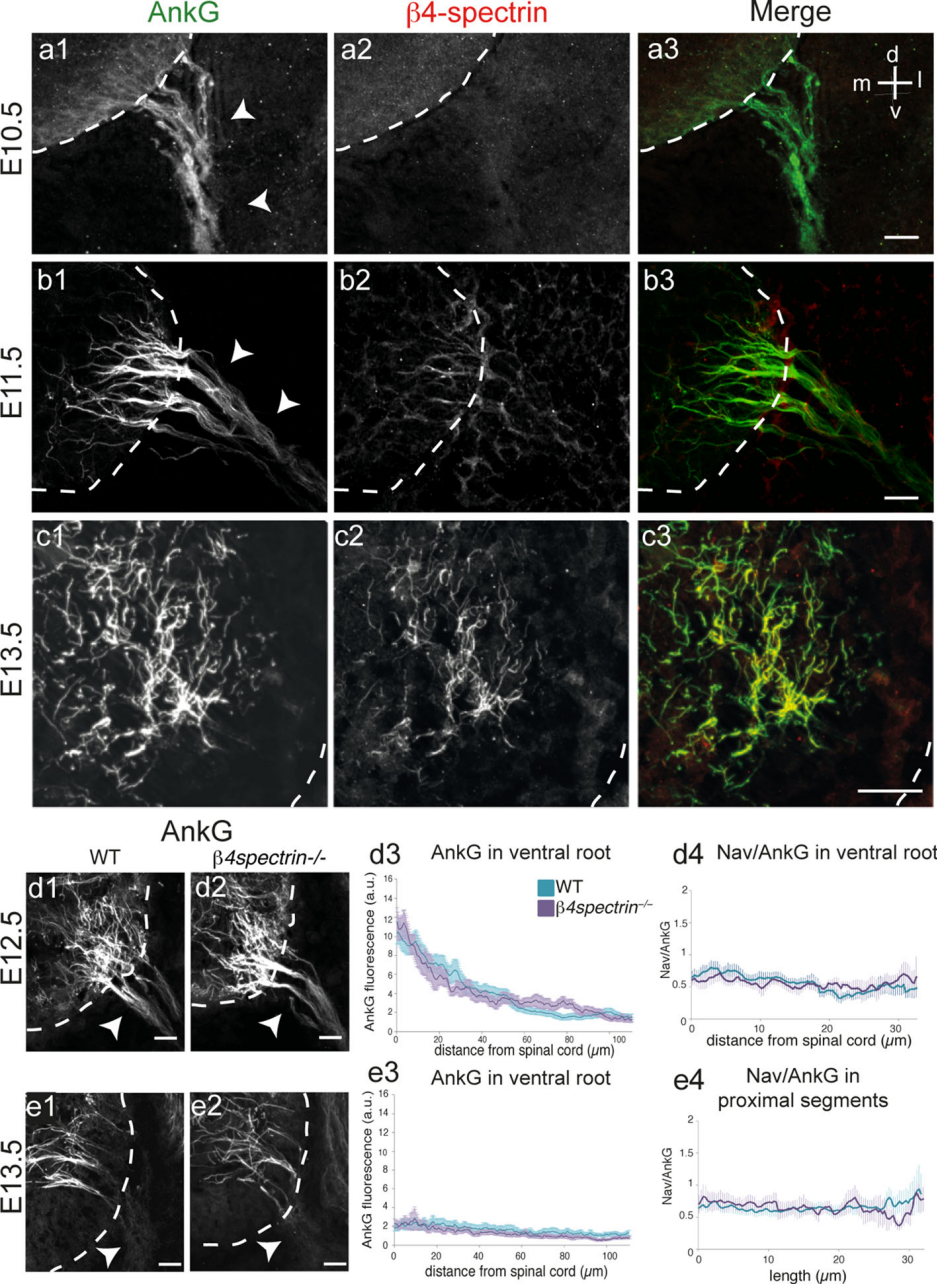
β4-spectrin expression starts being detected at E11.5 and is not required for the early AIS assembly. **a**–**c** Immunolocalization of AnkG (with rabbit anti-AnkG) (**a1**–**c1**) and β4-spectrin (**a2**–**c2**) was analyzed in spinal cord (delimited by *dashed lines*) and in ventral roots (*arrowheads*) at E10.5 (**a**), E11.5 (**b**) and E13.5 (**c**). **d–e** AnkG distribution (with rabbit anti-AnkG) was similar in *β4*-*spectrin* ^−*/*−^ (**d2**, **e2**) and WT embryos (**d1**, **e1**) both in the ventral spinal cord and in the ventral root at E12.5 (**d1**, **d2**) and E13.5 (**e1**, **e2**). AnkG IF profile along the ventral root (**d3**, **e3**, *n* = 3) in WT and *β4*-*spectrin* ^−*/*−^ embryos at E12.5 (**d3**) and E13.5 (**e3**). PanNav/AnkG IF intensities in the ventral root at E12.5 (**d4**, *n* = 3; 16 paths drawn) and within the spinal cord at E13.5 (**e4**, *n* = 3; 15 fibers measured). *Scale bar* represent 25 μm

We used two antibodies that both specifically recognize the two neuronal isoforms of β4-spectrin (Lacas-Gervais et al. 2004; Komada and Soriano 2002) and revealed the same expression pattern. At E10.5, when AnkG was already expressed all along MN axons (arrowheads in Fig. 7a1), β4-spectrin could be detected neither in the spinal cord nor in the ventral root (Fig. 7a2). β4-spectrin labeling appeared only at E11.5 and was strictly restricted to AnkG^+^ segments along MN axons (Fig. 7b). Because the labeling was very weak it could not be tracked along the entire length of AnkG^+^ segments; nevertheless, it could be observed both in the ventral root and within the spinal cord. At E12.5, β4-spectrin displayed a higher expression level and colocalized with AnkG in the spinal cord and along the ventral root (data not shown). At E13.5, when AnkG was restricted to spinal cord segments, β4-spectrin was similarly restricted and colocalized with AnkG (Fig. 7c). Thus, its developmental distribution closely matched that of Navs.

Given the role of β4-spectrin as a cytoskeleton linker protein for AnkG and AnkG-bound transmembrane proteins, we analyzed whether β4-spectrin was necessary for the proper developmental distribution of AnkG and in particular of Navs along motor axons. Loss-of-function experiments in cultured hippocampal neurons have suggested that β4-spectrin was not necessary to assemble the AIS (Hedstrom et al. 2007). However, its role in AIS assembly or maintenance in vivo was suggested from adult *β4*-*spectrin* ^−*/*−^ mice, where the AIS expression level of AnkG and Navs, compared to WT mice, was much lower in Purkinje cells (Komada and Soriano 2002) as well as in cortical neurons (Uemoto et al. 2007), where AnkG was also found to be improperly restricted to the AIS. We thus analyzed the early steps of AIS development, in particular the restriction of AnkG and Navs to the nascent proximal AIS in *β4*-*spectrin* ^−*/*−^ MNs at E12.5 (one day after β4-spectrin expression normally appeared), and E13.5 (Fig. 7d1–d3, E1–E3, respectively). Along ventral root axons, AnkG displayed the same distal-to-proximal restriction as in WT mice. In addition, the PanNav/AnkG IF along the ventral root at E12.5 and along the proximal segments in the spinal cord at E13.5 (Fig. 7d4, e4, respectively) did not reveal any difference between WT and *β4*-*spectrin* ^−*/*−^ mice.

These results indicate that β4-spectrin is not necessary for the proper spatio-temporal recruitment of AnkG and Navs during the early steps of MN AIS assembly in vivo.

### AnkG clustering and AnkB/α2-spectrin/β2-spectrin cytoskeleton

**Fig. 8:**
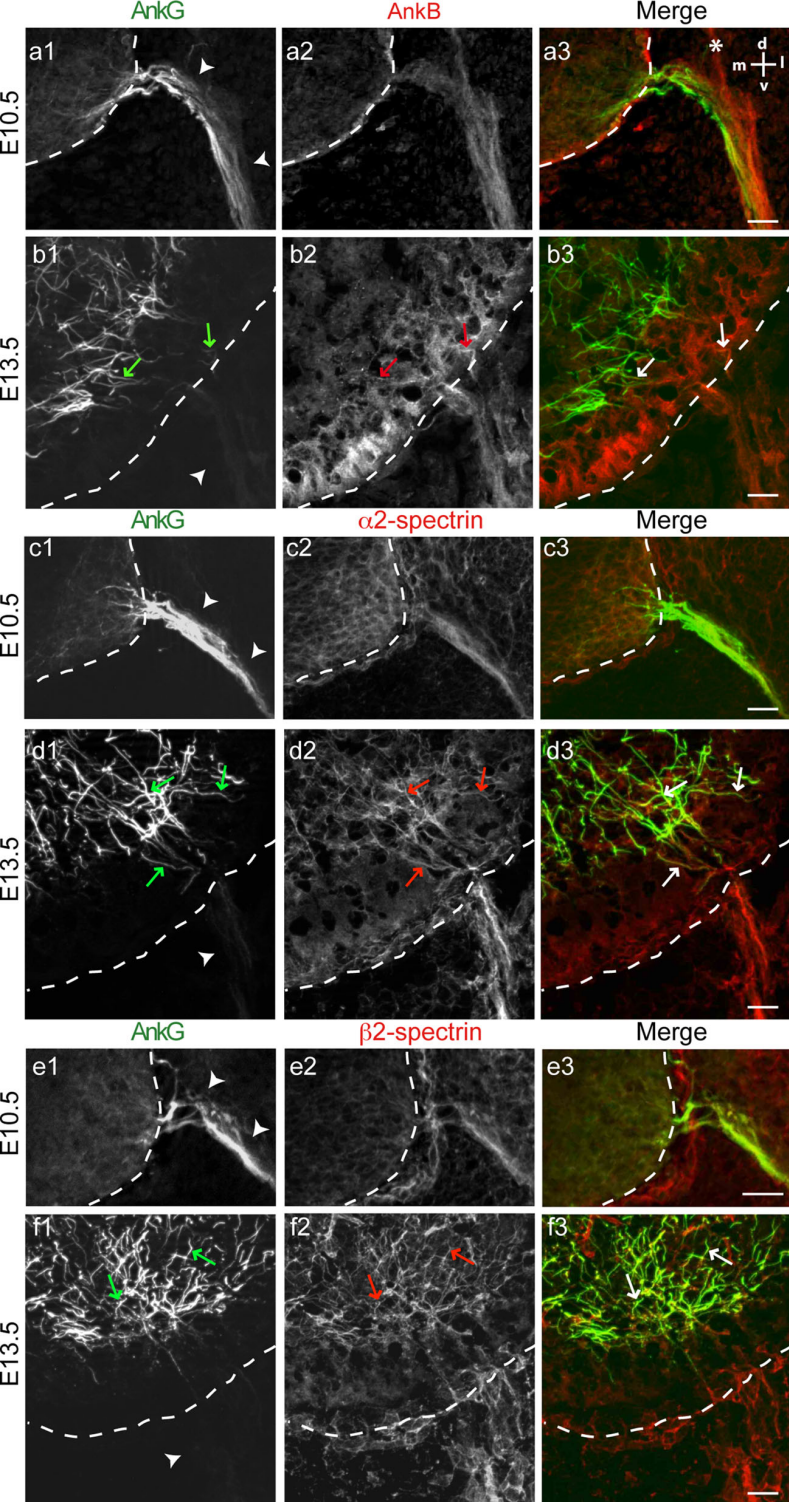
AnkB, α2- and β2-spectrin are not restricted to the distal part of motor axons. Immunolabeling of AnkG (with rabbit anti-AnkG) (**a1**–**f1**), AnkB (**a2**–**b2**), α2-spectrin (**c2**–**d2**) and β2-spectrin (**e2**–**f2**) in the spinal cord (delimited by *dashed lines*). At E10.5, AnkG (**a1**, **c1** and **e1**), AnkB (**a2**), α2-spectrin (**c2**) and β2-spectrin (**e2**) were expressed in the ventral root (*arrowheads*). At E13.5, AnkB (**b2**), α2-spectrin (**d2**) and β2-spectrin (**f2**) were expressed both along the ventral root and in some AnkG^+^ segments in the spinal cord (*arrows*). The *asterisk* in **a3** indicates the dorsal root. *Scale bar* represent 25 μm

AnkB is known to form a cytoskeletal protein complex with α2- and β2-spectrin at the paranodal junctions (Ogawa et al. 2006) and was very recently demonstrated to contribute to a distal sub-membranous complex which was proposed to form a barrier excluding AnkG from the distal axon (Galiano et al. 2012). We thus analyzed the distribution of these proteins at two stages of MNs’ development, at E10.5 and E13.5, i.e., before and after AnkG exclusion from the distal part of the axon. At E10.5, when AnkG was expressed all along motor axons (arrowheads in Fig. 1a1, c1, e1), we detected a weak and homogenous labeling of AnkB, α2- and β2-spectrin along the ventral root (Fig. 8a2, c2 and e2, respectively). At E10.5, AnkG, AnkB, α2- and β2-spectrin colocalized in the ventral root along MN axons. At E13.5, immunoreactivity for AnkB, α2- and β2-spectrin was stronger and could still be observed in the ventral root (arrowheads in Fig. 8b, d and f), whereas AnkG was already restricted to the proximal parts of motor axons in the spinal cord (Fig. 8b1, d1 and f1). Inside the spinal cord some AnkG^+^ segments also expressed AnkB (arrows in Fig. 8b). Likewise, we were able to detect AnkG^+^/α2-spectrin^+^ and AnkG^+^/β2-spectrin^+^ fibers (arrows in Fig. 8d, f, respectively). Thus, AnkB, α2- and β2-spectrin are expressed in motor axons but do not seem to be strictly restricted to the distal domain at least until E13.5.

### AnkG expression arises along with the first steps of axonogenesis and is a very early axonal marker

**Fig. 9:**
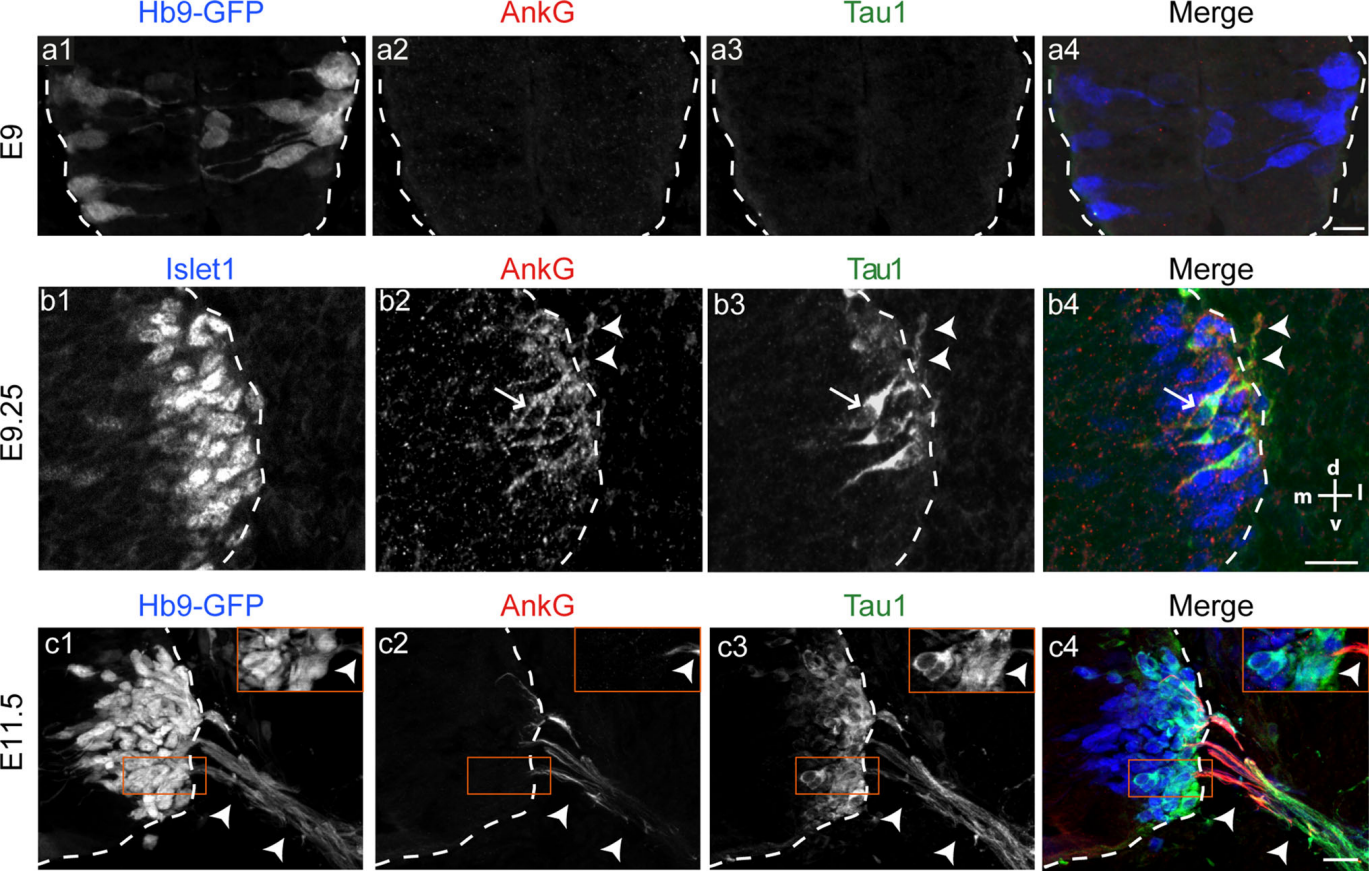
The early polarized distribution of AnkG makes it an early axonal marker in vivo. Immunolabeling of AnkG (with rabbit anti-AnkG) (**a2**, **b2**, **c2**) and Tau-1 (**a3**, **b3**, **c3**) in the spinal cord (delimited by *dashed lines*) and in the ventral roots (*arrowheads*) in E9 Hb9-GFP^+^ MNs (**a1**), in E9.5 Islet1^+^ MNs (**b1**) and E11.5 Hb9-GFP^+^ MNs (**c1**). AnkG displayed a polarized (axonal-only) distribution at E11.5 whereas Tau-1 was still expressed in axons and somatas. *Arrows* and *arrowheads* point, respectively, to a MN’s somato-dendritic and axonal compartment. *Scale bar* represent 20 μm

Since we found that AnkG is initially expressed in MNs as soon as they grew one process that emerged from the spinal cord, a process that is already or will become the axon, we compared the time course of AnkG distribution with that of the most widely used axonal marker Tau1 (Dotti et al. 1987). At E9, only a few HB9^+^ MNs could be found but neither AnkG nor Tau1 (Fig. 9a) was yet expressed in these MNs. At E9.25, when a few MNs started to grow a presumptive axon out of the spinal cord, AnkG started to be weakly detected in both axonal and somato-dendritic compartments (arrowheads and arrow, respectively, in Fig. 9b2) of some Islet1^+^ MNs. Similarly Tau1 (Fig. 9b3) was also detected at this stage in both compartments of the same MNs. Thus, at E9.25 expression of AnkG and Tau1 was not yet polarized. The polarized expression of AnkG (confined to the axon only) started for some neurons around E10.5 (Fig. 1c2) and was complete by E11.5 (Fig. 9c2), whereas Tau1 (Fig. 9c3) was still expressed in both axonal and somato-dendritic compartments of a large number of MNs at E11.5. Similar results were observed for SMI 312 (data not shown), another axonal marker (Ulfig et al. 1998; Matsuda et al. 2009). Thus, AnkG is a very early axonal marker in MNs.

## Discussion

The formation of a mature MN AIS is a long process, only a part of which is embryonic: the AIS expression of Kv1.1/1.2 as well as KCNQ2 potassium channels emerges in MNs during the first postnatal week (Devaux et al. 2004; Duflocq et al. 2011, unpublished data). Similarly, the shift of expression from Nav1.2 to Nav1.6 isoform occurs at the beginning of the MN myelination process (Duflocq et al. 2011). Our results show that AnkG, which is the AIS master organizer, is expressed much earlier (starting at E9.5), first along the whole axon and progressively only in its proximal part. The mean length of these proximal AnkG^+^ segments at E13.5 is similar to that of adult MN AISs (Duflocq et al. 2011). Furthermore, these segments contain functional Navs able to generate action potentials. Hence, we consider this early proximally-restricted AnkG^+^ segment as an ‘AIS’, even though it is not yet fully mature.

### AnkG clustering at the proximal part of the axon

A major result of this work is to show that the assembly of the AnkG^+^ segment at the proximal part of the motor axon is the consequence of two successive processes: (1) AnkG invades the whole axon including its defasciculating terminal; (2) AnkG is progressively restricted to the initial part of the axon. During the first phase, the axonal distribution of AnkG is homogenous with a proximal limit whose distance to the soma is variable. This clear-cut proximal limit was detected at the very beginning of AIS formation.

Previous data on the time course of AnkG appearance in vivo were obtained in developing Purkinje cells. AnkG^+^ and β4-spectrin^+^ segments were first detected at P2 but the analysis of embryonic stages was not possible at that time due to the difficulty of identifying Purkinje cell axons in embryos (Jenkins and Bennett 2001).

Recently, the emergence of AnkG expression was analyzed in developing cortical neurons in vivo (Galiano et al. 2012). AnkG expression was not detected in migrating neurons at E18 whereas at P1, when most neurons had reached their final destination, they exhibited AnkG immunoreactivity in their proximal axon. In these neurons, AnkG never invaded the whole axon; the distal end of AnkG staining always occurred at a fixed distance from the soma and this was due to the presence of an AnkB/α2-spectrin/β2-spectrin cytoskeleton in the distal axon, limiting AnkG to the proximal region (Galiano et al. 2012). These observations differ from our results in MNs: (1) In MNs, the establishment of the AIS proximal limit precedes that of the distal limit; (2) No distal segregation of the AnkB/α2-spectrin/β2-spectrin cytoskeleton was detected in MNs even when the restriction of AnkG was complete at E13.5. Furthermore, at this developmental stage many axonal segments coexpressed AnkG and AnkB. Thus, the mechanisms controlling the emergence of AnkG expression appear to be different in MNs and cortical neurons. From our results, it can be assumed that the formation of the nascent AIS in MNs is the result of two distinct and consecutive processes: (1) the recruitment and stabilization of AnkG along the axon from a proximal point that constitutes the proximal limit of the nascent AIS; and (2) the exclusion of AnkG from the distal axon. We thus suggest that depending on the relative timing of these two processes the distal axon may or may not transiently express AnkG. This could explain why cortical neurons do not express AnkG in their distal axon in contrast to MNs. Moreover, the level of immunostaining of AnkB, α2-spectrin and β2-spectrin was low in developing MNs and thus the immunohistochemical approach alone is not sufficient to exclude a role of AnkB/α2-spectrin/β2-spectrin cytoskeleton in the AnkG segregation in the proximal part of MN axons as described in cortical neurons. The fact that β4-spectrin is not involved in the proximal restriction of AnkG in MNs suggests that at least this process does not depend on the ability of AnkG to bind the actin cytoskeleton through β4-spectrin.

At E11.5 and E13.5, i.e., before and after AnkG was restricted to the proximal axon, the protein was strictly restricted to the sub-membranous compartment as shown by electron microscopy. It was very recently shown that AnkG is S palmitoylated at a conserved cysteine residue, required for its membrane association and the recruitment of AnkG and Nfasc at the AIS of cultured hippocampal neurons (He et al. 2012). This S palmitoylation could be similarly involved in the membrane association of AnkG in developing motor axons. However, AnkG could also interact with one or several membrane proteins, which stabilize it in the sub-membranous region and one such candidate is Nfasc.

### The AIS components emerge sequentially in vivo

Our results also indicate that, during the embryonic development of MNs in vivo, the major AIS proteins do not appear simultaneously; first AnkG and Nfasc emerge, then Navs and β4-spectrin start being expressed. This is in contrast to results obtained in vitro: in cultured MNs dissociated from E14 rat spinal cords, Navs were restricted to the proximal region of the axon from the very first day in culture (Alessandri-Haber et al. 1999). Likewise, in cultured hippocampal neurons, even though the expression of AnkG appears later (only from day 3 in culture: Song et al. 2009), AnkG seems to accumulate immediately at the AIS and no progressive restriction of its distribution could be observed. Furthermore, no sequential emergence of the different AIS proteins has been reported in vitro. One hypothesis to explain these differences from our in vivo observations is that these hippocampal neurons as well as cultured MNs have been dissociated, in preparation for culture, after axonogenesis had started in vivo. At least some of the building program of the AIS may thus have been triggered and the observations made in vitro could thus result, at least partly, from axonal regeneration rather than axonogenesis per se (Barnes and Polleux 2009). This underlines the importance of observing these events in vivo.

From the onset of their expression, Nav channels are colocalized with AnkG and Pan Nav/AnkG IF ratio is constant throughout the axon. This is in good agreement with the fact that Navs are stabilized by their direct interaction with AnkG through their specific interacting sequence previously described (Lemaillet et al. 2003; Garrido et al. 2003). Finally, the expressed Navs are functional since action potentials were detected as soon as at E12.5, a stage when AnkG and Navs are not completely restricted to the AIS. Furthermore, at this stage the connection of motor axons with muscle was not completed. The spontaneous action potentials that were detected could thus be involved in the maturation of spinal neuronal networks.

### AnkG expression and axonogenesis

Our results show that AnkG was not detectable in bipolar migrating MNs and was expressed in the first hours of axon life, the axon being characterized as the process that extends out of the spinal cord (Wentworth and Hinds 1978). The axonal-restricted targeting of AnkG occurred earlier during development than that of the axonal markers Tau1 and SMI312 demonstrating that AnkG is a very early axonal marker. This also suggests that AnkG could be involved in the specification of axons as shown in *C. elegans* where Ankyrin together with CRMP organize microtubule asymmetry and axon-dendrite sorting (Maniar et al. 2011). In mice with a deletion of a cerebellum-specific AnkG isoform (Zhou et al. 1998) although cerebellar axons progressively lose their identity from the second week of postnatal life, axons appear normal beforehand, suggesting that their formation is not altered (Sobotzik et al. 2009). Similarly, after in utero electroporation of an AnkG-specific shRNA in rat embryos (at E14), electroporated cortical neurons still had an axon that crossed the *corpus callosum*. These results indicated that AnkG was not necessary for axon specification, while it was required for maintaining neuronal polarity at later stages (P28) (Galiano et al. 2012). However, in both experiments it cannot be excluded that a transient expression of an early Ankyrin isoform occurs, which could play a role in axonogenesis in both cell types.

We conclude that the formation of nascent AISs in MNs is the result of two distinct and consecutive events concomitant with the specification of MN axons. The first event consists of the specific and early targeting of AnkG and Nfasc into the growing axon. At this early stage, the proximal limit of the AIS is established. The second event is the restriction of AnkG and associated membrane proteins at the initial segment and stabilization of the distal limit of the AIS.

## Electronic supplementary material

Below is the link to the electronic supplementary material.

Supplementary material 1. Fig. S1 Anti-AnkG antibodies. **a**, **b** Immunolabeling with polyclonal rabbit (**a1**) and guinea pig (**b1**) anti-AnkG detected in E16.5 ventral spinal cord slices was suppressed after pre-incubation with the antigenic peptide (**a2** and **b2**). **c** Western Blot on protein extracted from E16.5 spinal cord with rabbit anti-AnkG revealed protein bands that correspond to 480 and 270 kDa AnkG isoforms. With protein extracted from adult liver, no signal was observed. **d** Immunolabeling with polyclonal rabbit (**d1**), guinea pig (**d2**) and monoclonal mouse (**d3**) anti-AnkG strictly colocalized in ventral spinal cord at E16.5. *Scale bar* represent 10 μm (TIF 1.49 MB).
