# Supplementary figures and images for: In vivo assembly of the axon initial segment in motor neurons
Source: Brain Struct Funct. 2013 Jun 2;219(4):1433–50. doi: 10.1007/s00429-013-0578-7 (PMC4072062; doi:10.1007/s00429-013-0578-7)

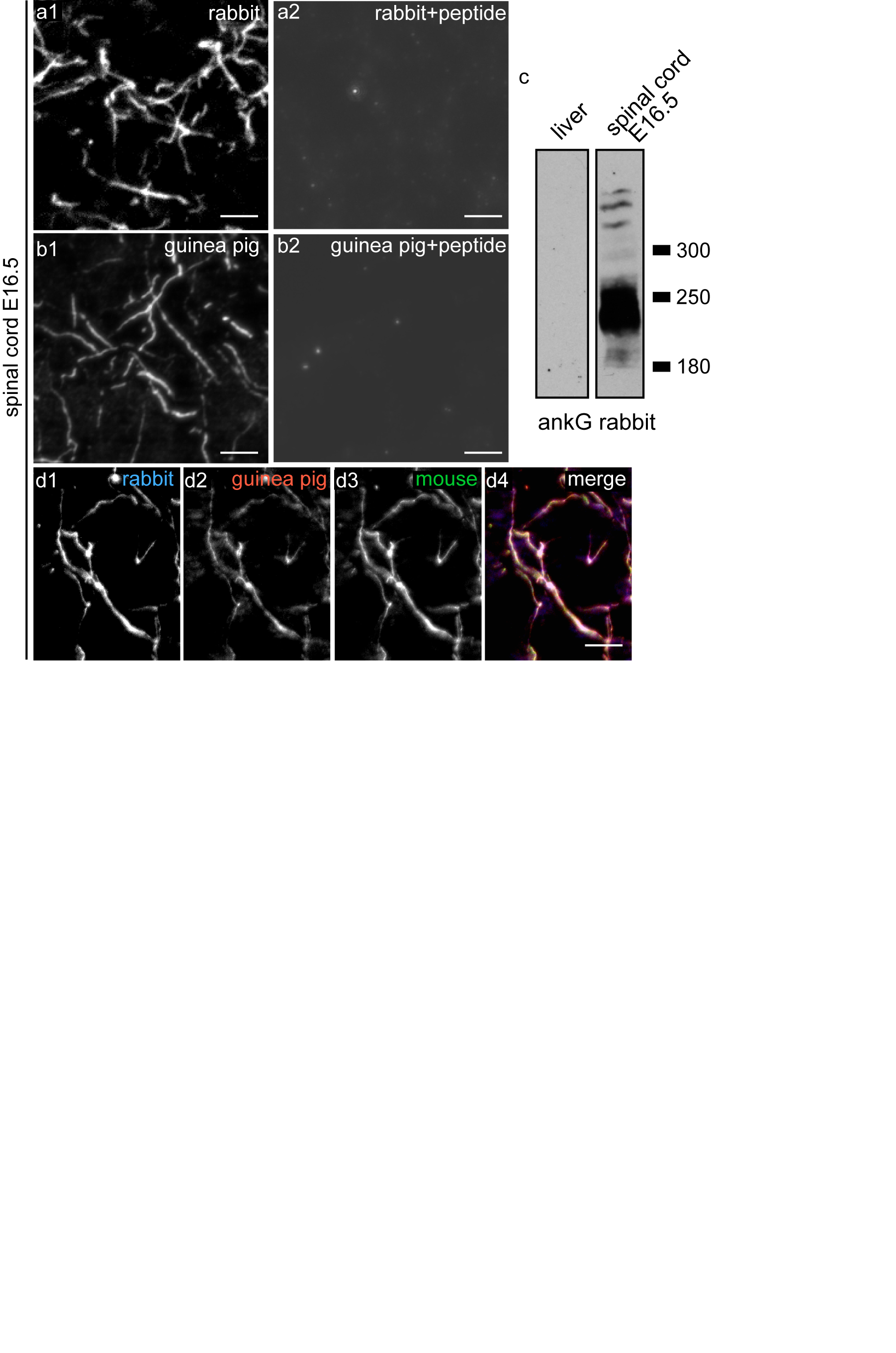

Supplement: Supplementary file 1 — Supplementary material 1. Fig. S1 Anti-AnkG antibodies. a, b Immunolabeling with polyclonal rabbit (a1) and guinea pig (b1) anti-AnkG detected in E16.5 ventral spinal cord slices was suppressed after pre-incubation with the antigenic peptide (a2 and b2). c Western Blot on protein extracted from E16.5 spinal cord with rabbit anti-AnkG revealed protein bands that correspond to 480 and 270 kDa AnkG isoforms. With protein extracted from adult liver, no signal was observed. d Immunolabeling with polyclonal rabbit (d1), guinea pig (d2) and monoclonal mouse (d3) anti-AnkG strictly colocalized in ventral spinal cord at E16.5. Scale bar represent 10 μm (TIF 1.49 MB). [file 429_2013_578_MOESM1_ESM.tif]
